# Risk Assessment-Oriented Design of a Needle Insertion Robotic System for Non-Resectable Liver Tumors

**DOI:** 10.3390/healthcare10020389

**Published:** 2022-02-18

**Authors:** Bogdan Gherman, Nadim Al Hajjar, Paul Tucan, Corina Radu, Calin Vaida, Emil Mois, Alin Burz, Doina Pisla

**Affiliations:** 1 CESTER—Research Center for Industrial Robots Simulation and Testing, Technical University of Cluj-Napoca, Bulevardul Muncii Street, No. 103-105, 400641 Cluj-Napoca, Romania; bogdan.gherman@mep.utcluj.ro (B.G.); paul.tucan@mep.utcluj.ro (P.T.); calin.vaida@mep.utcluj.ro (C.V.); alin.burz@mep.utcluj.ro (A.B.); 2 “Prof. Dr. Octavian Fodor” Regional Institute of Gastroenterology and Hepatology Cluj-Napoca, Croitorilor Street, No. 19-21, 400162 Cluj-Napoca, Romania; na_hajjar@yahoo.com (N.A.H.); drcorinaradu@gmail.com (C.R.); drmoisemil@gmail.com (E.M.)

**Keywords:** medical robotics, risk assessment, cancer treatment, failure modes analysis, robot design, needle insertion, fuzzy logic

## Abstract

Medical robotics is a highly challenging and rewarding field of research, especially in the development of minimally invasive solutions for the treatment of the worldwide leading cause of death, cancer. The aim of the paper is to provide a design methodology for the development of a safe and efficient medical robotic system for the minimally invasive, percutaneous, targeted treatment of hepatocellular carcinoma, which can be extended with minimal modification for other types of abdominal cancers. Using as input a set of general medical requirements to comply with currently applicable standards, and a set of identified hazards and failure modes, specific methods, such as the Analytical Hierarchy Prioritization, Risk Analysis and fuzzy logic Failure Modes and Effect Analysis have been used within a stepwise approach to help in the development of a medical device targeting the insertion of multiple needles in brachytherapy procedures. The developed medical device, which is visually guided using CT scanning, has been tested for validation in a medical environment using a human-size ballistic gel liver, with promising results. These prove that the robotic system can be used for the proposed medical task, while the modular approach increases the chances of acceptance.

## 1. Introduction

Liver cancer is a widely spread disease, currently growing all over the world [1]. Statistics [2] show that, by 2025, the cancer incidence will be more than 1 million annually. Hepatocellular carcinoma (HCC) is by far the most prevalent primary liver cancer; 90% of it arises from cirrhosis, hepatitis B virus being the most prominent risk factor, accounting for about 50% of all cases [3]. Non-alcoholic steatohepatitis (NASH) and nonalcoholic fatty liver disease (NAFLD) are slowly becoming one of the most relevant causes of HCC [4]. The treatment of HCC is more than often complicated and complex, needing a multidisciplinary team for its implementation, especially if the case is complicated. It is highly dependent on the quality of evidence provided, the detection stage [5,6,7,8], and on local development and available resources. It is well known that the surgical approach is most of the times the best solution for long-term survivability [9], being focused on two main directions: liver resection and liver transplantation, [10,11]. Liver transplantation can be performed in certain conditions, specified usually by the University of California San Francisco (UCSF) criteria, but it has a high recurrence incidence [12,13].

Despite the effectiveness of the surgical approach, only ~30% of the cases can be subject of resection, the cancer detection being in an advanced stage. Therefore, other approaches with focus on targeted therapies, which minimize the effects on the whole body have been developed and used. Ablation (e.g., radiofrequency (RF), microwave, percutaneous ethanol injection, cryotherapy, high-intensity focused ultrasound ablation) is suitable for patients with BCLC Stage 0 and sometimes Stage A HCC, [14]. Transarterial Chemoembolization (TACE) is one of the most popular non-surgical procedures for the treatment of HCC, being especially suitable for the patients with BCLC Stage B [15]. Radiation therapy, classified as external radiation and internal radiation is another popular choice, sometimes used in conjunction with TACE or even surgery. External radiotherapy is delivered from the outside of the body by aiming beams of radiation towards the tumor in a minimally invasive way. Internal radiotherapy is performed by delivering radiation particles (Y-90, iodine-131 monoclonal antibodies, radioactive lipiodol, iodine-125) into the tumor using various channels, e.g., interstitial implantation, portal vein implantation, inferior vena cava implantation, portal vein tumor thrombi, etc. [16]. External beam radiation is mostly used for patients in CNLC Stage Ia and sometimes with Stage Ib HCC. Similarly, internal radiotherapy is mostly recommended for patients with CNLC Stage Ia HCC and a sometimes for patients with Stage Ib, and in certain cases and in conjunction with other treatment therapies (SBDR or TACE), it can be used for palliative purposes, preserving the patient’s life quality.

As observed, the reasons behind choosing one type of treatment over the other are strongly dependent on the HCC CNLC stage and the overall condition of the patient (i.e., comorbidities or age), but also on economic criteria, which is the case in less developed countries or regions. For this purpose, especially when resources are limited, the team of doctors will choose the best therapy from the available ones within the realistic conditions.

Since most important clinics and hospitals are equipped with computer tomography machines (CT) and/or Magnetic resonance imaging machines (MRI), researchers have developed remotely guided minimally invasive systems that can be used in conjunction with these machines, which can be used for visual control and guidance. When metallic parts (needles) are used, the CT is the only option for a visual control of the device. Since the solutions dedicated for the specific treatment of HCC involving needle insertion (e.g., brachytherapy) are very rare, the study of the current existing systems targets broader horizons, including mainly the CT-guided devices, since these have revealed important aspects which have been considered within the present research. In [17], the authors have proposed a robotic system (Zerobot) designed to fit the CT bore and percutaneously insert a needle on a linear trajectory in a minimally invasive way. It has 6 Degrees of Freedom (DoFs)—4 translations and 2 rotations in a serial architecture and an operator user interface which is used to control the needle position and orientation. The needle placement measured accuracy has been very similar to manual insertion (~1.6 vs. 1.4 mm), as has been also the insertion time, but the radiation exposure of the physician (human operator) during the insertion procedure has been 0, compared to the manual procedure (~5.7 μSv).

Another system, the AcuBot [18], has been developed for RF ablation and is guided using the CT, having 6 DoFs used for position and orientation as well as needle insertion. It has been used in validation tests on kidney and spine. Innmotion [19] is a CT and MR-compatible robotic system designed for instrument guiding consisting of a passive rotational 1 DoF joint for manual position and a 6 DoFs pneumatically actuated robot for percutaneous biopsy, drainage, and tumor ablation within the thoracic and abdominal areas. The Mitsubishi RV-E2 [20] is a general-purpose articulated robot having 6 DoFs has been tested to drive a needle to reach a lung nodule in phantom tests using a specially designed gripper. MAXIO [21], produced by Perfint Healthcare Pvt. Ltd., is a commercially available robotic system designed for the positioning and orientation of an instrument to assist in manual insertion of straight needles and probes in CT guided percutaneous procedures. It can be used for the thorax, abdomen, and pelvis. XACT robotics Ltd. [22] have proposed a robotic system for needle guidance in percutaneous interventions using CT guidance through using a specially designed table which is placed on the patient’s abdominal cavity (so it naturally accounts for patient’s breathing), being remotely steered with an EM tracker. Up to now, to the best of our knowledge, it did not obtain the CE mark. In [23], the authors have developed a robotic system using ultrasound guidance and optical tracking to accurately position the end-effector of a KUKA LBR 7 R800 articulated arm for further use within ultrasound-guided radiation therapy. Solutions have been also developed to serve the automatic recognition of various features [24,25]. It also has breathing compensation. The main drawback in most of these systems is related to the high cost for the development and purchase and the necessity of additional personnel, all of which may not always be very profitable.

The paper focuses on the development of a robotic solution for the minimally invasive treatment of HCC. Using known design methods, the knowledge and experience of the engineers specialized in the development of medical devices and of medical doctors led to the definition of a set of requirements based on which a modular robotic system for percutaneous needle insertion using the internal radiation approach (brachytherapy) under CT guidance, has been designed, as an alternative to classic manual needle insertion. The modular approach brings an important advantage: the potential to reuse existing elements for other medical applications with minimal modifications. Section 2 of the paper presents the methods used for the efficient and safe design of a robotic system, starting from the medical requirements, their analysis, a risk assessment based on an experienced team of engineers and doctors specialized in cancer treatment, and a fuzzy-based FMEA process. The experimental model of the proposed robotic system is presented in Section 3, an analysis of the proposed methodology is performed in Section 4, and some conclusions are drawn in Section 5.

## 2. Materials and Methods

**Figure 1:**
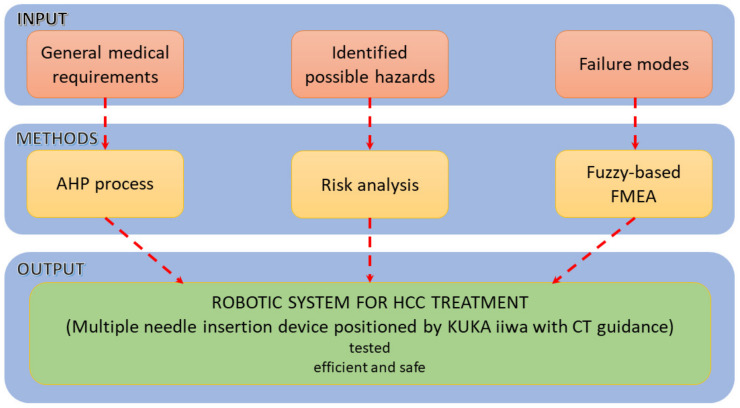
The methodology used for the development of the percutaneous needle insertion robotic system.

The research methodology is graphically explained within Figure 1. Based on information related to medical general requirements regarding the approached HCC treatment method, the identified possible hazards which may be encountered during the procedure and the different identified failure modes, different methods (AHP, Risk analysis and fuzzy-based FMEA) are used to help provide a suitable design for the proposed medical device.

To comply with the ISO 11608 and the accuracy requirements of about 2.5 mm inside the patient’s body [26], a set of requirements have been defined.

### 2.1. General Requirements

1. The robotic system should be able to position the needle within a very tight space in the CT bore, above the patient’s abdominal cavity (functional requirement Func-01).

Most of the traditional CT machines for diagnosis vary between 650–700 mm, which is why wider CT machines featuring bores between 800–1000 mm have been introduced [27]. Nevertheless, within the development phase, the authors need to consider that the existing resources within hospitals and clinics across Europe consist in the classical sizes CT bores and design the needle insertion accordingly.

2. The robotic system should be able to provide a very accurate orientation of the needle inside the CT bore (functional requirement Func-02).

The needle insertion is preferably to be performed within the CT plane (the scanned sections of the patient’s body). However, sometimes this cannot be achieved due to safety reasons [28,29]. Handheld manual insertion can become challenging when a certain angle (with the CT plane) needs to be maintained during the procedure, due both to the initial set of the needle as well as during and after the CT scans are performed. The reason is that the needle needs to be released (remaining within the patient’s body) and because only small portions of the needle are observed during scanning.

3. The robotic system should be able to position accurately the needles in a predetermined array (functional requirement Func-03).

The radiation seeds (e.g., Iodine 125) need to be precisely placed within the tumor using linear arrays (matrix), thus being able to maintain the correct spacing between them which helps to correctly determine the dosimetry towards an increased efficiency [30].

4. The robotic system should be stiff enough to ensure a high precision (design requirement Des-01).

The accuracy of a robotic system (mechanism) is strongly related to its stiffness [31]. Nevertheless, the overall stiffness must be assessed according to the working conditions, especially the dynamic requirements of the application. Generally, the working velocities and accelerations in medical robotics are low compared to the industrial field, which diminishes the overall importance of this aspect. Even so, the static stiffness of the device is important, and it may be a challenge due to the tight space (leading to rather small size components) and long needles.

5. The robotic system should have a modular architecture for a broad variety of clinical applications (design requirement Des-02).

Modularity is a common requirement nowadays [32,33,34], since it spurs interoperability and standardization, all of which will cut costs, shorten the development time, and provide better maintenance for a longer life span [35] of the medical devices, [36]. Since the same tool or tools can be reused by the client, the manufacturers (and developers) may consider various platforms that, with minimum adjustments can be used for various medical applications, with similar user interfaces and operating environments, which is an incentive for the adoption of the device.

6. The needle insertion device should be sterilizable (design requirement Des-03).

The needle insertion medical device and all its components need to be sterilized before they can be used in the operating room. Popular types of sterilization include steam, dry heat, vaporized hydrogen peroxide, gamma, X-ray, or ethylene oxide [37]. The most common, by a considerable margin seems to be steam sterilization using the autoclave. Because of the electric circuitry sensibility to high temperature and humidity, there are two ways in which sterilization can be achieved:

- remove all electronics (including actuation motors) by designing it in such a way that it allows an easy connection/disconnection and removal.
- use sterilizable electronic equipment.

Depending on the complexity of the device or other requirements, a manufacturer can choose one method over the other.

7. The robotic system should account for patient breathing (control requirement—Con-01).

During inhalation, the diaphragm contracts and therefore it pushes the content of the abdominal cavity towards the inferior region, while the intercostal muscles expand the rib cage. During exhalation, the process is reversed, so the abdominal cavity organs are moving back to their original position (upwards), while the rib cage diminishes its volume. Authors have analyzed various solutions to solve this issue: motion (tumor) chasing [38] and motion gating [39]. Tumor chasing in real time is a very demanding task and is difficult to be achieved correctly. It requires the tumor identification, anticipation of the tumor motion, and the reposition of the medical device accordingly. Motion gating implies an external device which monitors the patient’s respiratory cycle, so that the medical intervention is achieved during specific time intervals: during the expiratory pause, when the tumor is (at least theoretically) stationary for a period of about 0.5 s.

8. The robotic system should account for the needle deflection (control requirement—Con-02).

This task can be achieved mostly using visual means (US, CT, MRI). Most basic needle types include blunt, beveled, conical, Sprotte, Franseen, or Tuhoy [40]. Among the parameters that influence the needle–tissue interaction are the needle geometry (the tip shape, diameter, and length), the insertion velocity, its law of motion during insertion and the elastic properties, which vary according to the penetrated organs and tissues. Except the steerable needles [41], the needle deflection is very difficult to be controlled; thus, if the needle deflects too much, it must be removed, the trajectory recalculated, and the needle reinserted on a new path.

9. Seamless integration within the clinical workflow (control requirement—Con-03).

The implementation of a new medical device should account for the existing medical protocols, which should be kept in place. The integration of the equipment has multiple benefits: efficiency, easiness, familiarity, and easier maintenance, all of which lead to an increased adoption potential and thus contributes towards the clinical and commercial success of the equipment [42].

**Figure 2:**
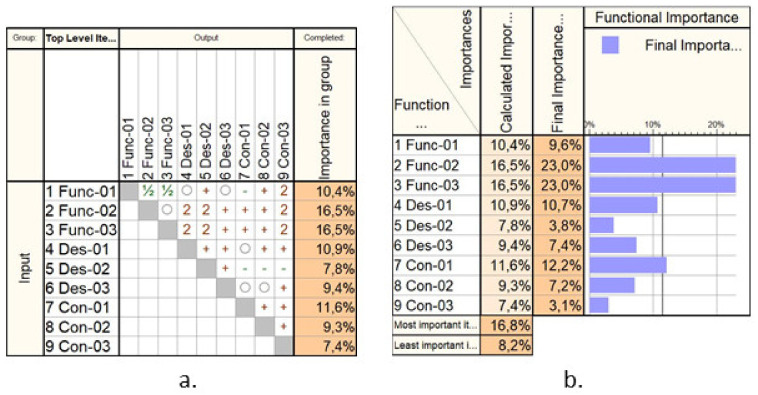
The Analytical Hierarchical Prioritization of the needle insertion device: (**a**) the correlation matrix; (**b**) the calculated importance.

Figure 2 presents a hierarchical prioritization of the requirements, performed using Qualica Quality Function Deployment [43] to assess the most important ones, which should be given appropriate attention within the design of the medical device. By a considerable margin, the Func-02 and Func-03 show the highest importance degree since these are the main functionalities of the needle insertion device. Of course, other parameters must be considered as well, such as the capability of ensuring various needle insertion velocities or a reduced payload, but these characteristics have been considered as default and did not affect the performed prioritization.

### 2.2. The Solution

**Figure 3:**
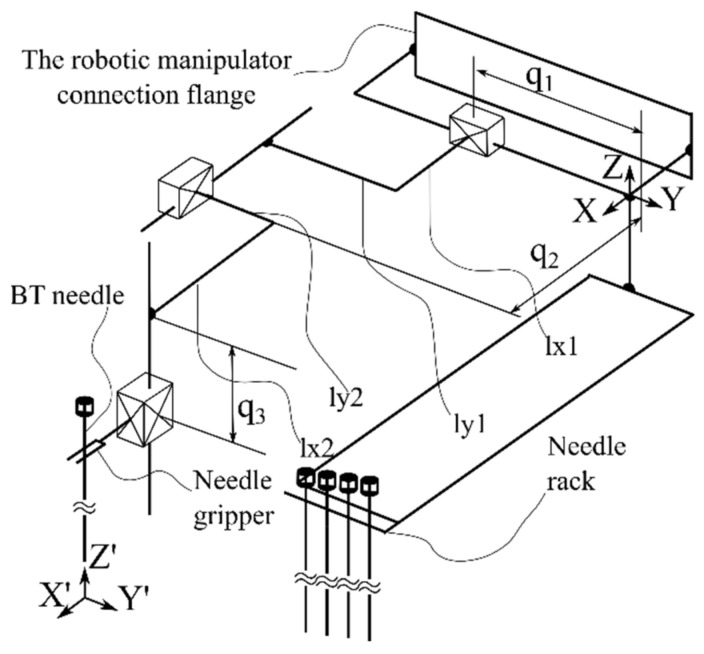
The Multiple Needle Insertion Device (MNID).

Based on the presented requirements, a demonstrator was built in a form of a collaborative robotic arm equipped with an innovative needle insertion module mounted on a mobile platform that enables the synchronous motions of the robot with the CT mobile couch to enable real-time needle position monitoring during the procedure. Figure 3 presents the kinematic scheme of the needle insertion device, which can insert multiple needles using just the initial position and orientation of the Multiple Needle Insertion Device (MNID), [44,45]. This comes as a requirement due to the need to position the needles in an array (Func-03) and to avoid collision with previously inserted needles. Thus, the MNID has 3 DoFs and a Gantry architecture which can position the needle in the XOY plane (2 DoFs) and perform the insertion along the OZ axis (1 DoF). The MNID has also a 1 DoF actuated robot gripper, designed to grip the needles, drive them to the insertion point and insert them on a linear trajectory. Trapezoidal screw–nut mechanisms have been foreseen to perform all the required motions of the MNID.

**Figure 4:**
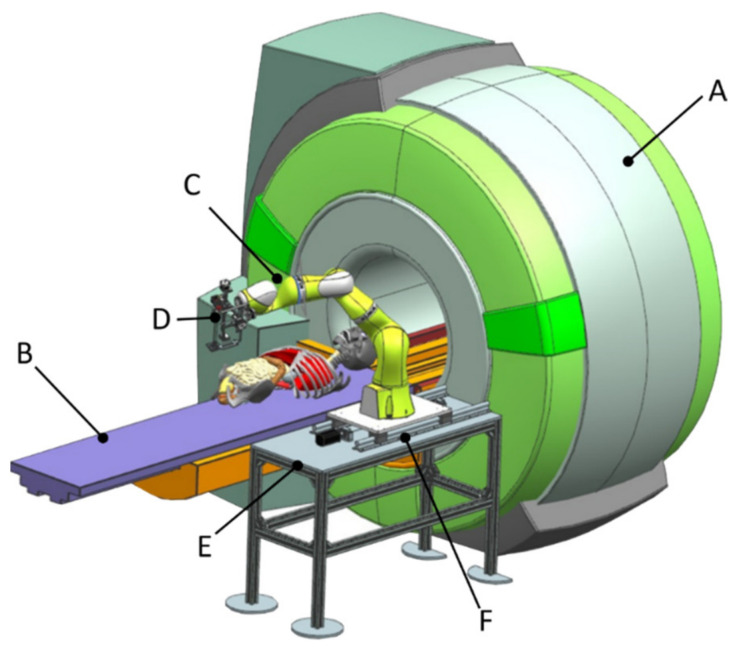
The complete robotic setup including the MNID and the KUKA iiwa.

The complete conceptual solution is presented in Figure 4, where the MNID is positioned using the KUKA iiwa LBR R800 robotic arm. Although the KUKA iiwa does not meet the required standards to be used in medical applications, the similarity level compared to its sibling, the KUKA LBR Med [46], makes the replacement very easy when looking forward for future tests with patients. The system’s architecture is presented within the medical environment and three main components:

1. Component 1 (A—CT scanner and B—external axis with 1 translational DoF)
2. Component 2 (C—robotic system and D—MNID)
3. Component 3 (E—stand and F—mobile platform that moves on a trajectory parallel with the moving table (couch) of the CT)

Components E and F (the 1 DoF external axis) has been designed to translate the robotic system (with MNID attached) along and together with the CT couch when performing the visual control CT scans. A detailed description of the system’s experimental model will be given in Section 3.3 of the paper.

### 2.3. Risk Analysis

**Figure 5:**
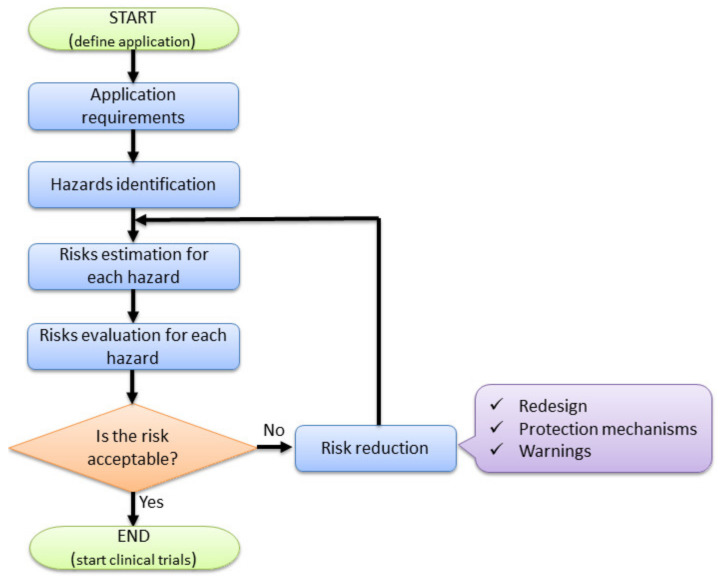
Risk-based design of the MNID robotic system.

ISO 12100:2010 “Safety of Machineries—General principles for design—Risk assessment and risk reduction” is the standard which provides the required means (principles and methodology) required to analyze and provide the safety design of the machines [47]. A simplified and adapted to the current application flow chart regarding the risk analysis is provided in Figure 5, underlining the main steps within the risk management of the MNID robotic system: definition of application (with specific requirements), hazard identification, risk estimation, risk evaluation, and finally, risk reduction.

Various types of hazards have been identified: mechanical, electrical, thermal, radiation, vibration, ergonomic, or a combination of them.

Mechanical hazards:

M1: Needle deflection. This may lead to very dangerous situations: important blood vessels penetration (i.e., hepatic artery and the portal vein, which could lead to massive hemorrhage) or missing the target points within the tumor.

M2: Collision between the MNID and the patient’s abdominal cavity. The robotic instrument may collision with the patient abdominal cavity before or during needle insertion procedure.

M3. Collision with the CT. The MNID or the robotic arm may collision with the CT elements, inside the CT bore.

M4. The robotic system is unable to follow the CT couch during CT scanning procedure. In this case, a delay between the two moving sub-systems may cause a reposition of the needle inside the abdominal cavity, which may lead to an inaccurate positioning of the needle or unwanted accidents (the needle might harm the internal organs).

M5. Poor needle placing accuracy. This may happen due to several reasons: unsecured positioning within the needle rack, patient breathing or undetected needle deflection.

M6. Faulty needle gripping. Due to various reasons (i.e., unsecured needles position within the rack), the needle may not be properly held by the gripper, causing a faulty placing, or slipping during insertion.

Electrical hazards:

E1: Patient electrocution. Due to improper grounding or wire isolation, there is risk of patient electrocution.

E2: Sensor malfunction. Included sensors: distance sensor—the robotic system will not be able to follow the CT couch; proximity sensor—the initialization procedure may fail.

E3: Risk of short circuit.

Thermal hazards:

T1: The patient may suffer burns by meeting overheating parts of the robot.

Radiation hazards:

I1: The patient will be irradiated (electromagnetic—X-rays) during each CT scan. All scans will be performed locally, targeting only the lesion area and the needle.

Vibration hazards:

V1: Poor needle accuracy placement due to vibration effects.

V2: Patient may be harmed during needle insertion due to uncontrolled vibrations of the MNID.

Ergonomic hazards:

ER1: The patient position on the CT couch may change due to bad initial positioning.

**Figure 6:**
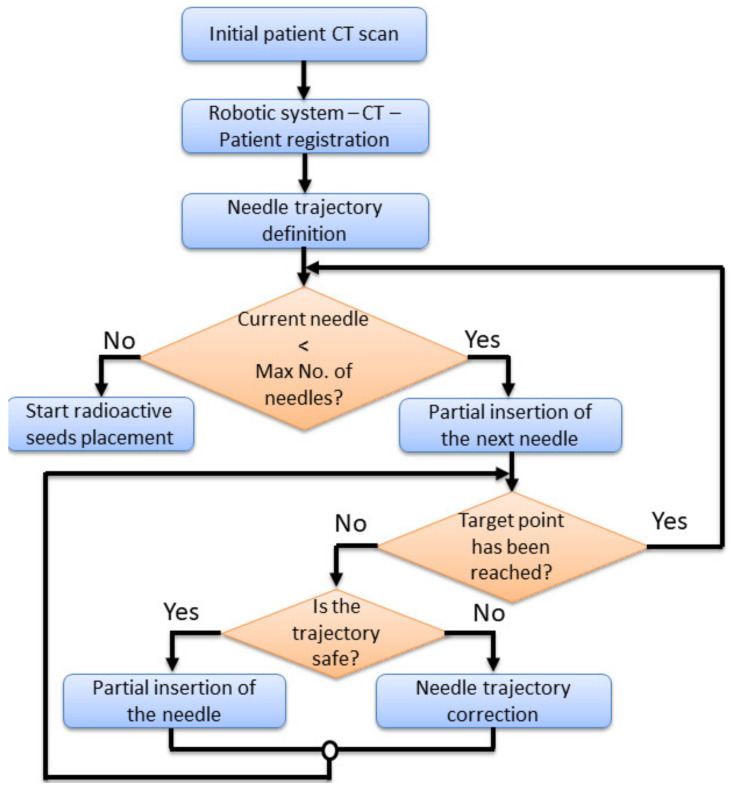
The needle insertion procedure flow chart using the MNID.

The limits of machinery define the conditions in which the robotic system is operated. The robotic system needs to be able to insert percutaneously multiple needles up to a predetermined target point, under the visual guidance of the CT. Figure 6 presents a needle insertion robotic-assisted protocol with the following main steps:

- Preplanning. Before performing the insertion procedure, based on an initial scan, a preplanning procedure is required, to define the safe needle insertion trajectories.
- Tumor’s registration. The patient, CT and the robotic system must run a registration procedure, where the position of the tumors is exactly defined within the robotic system coordinates. This is achieved using a set of metal markers (steel balls) fixed placed on the patient’s body.
- Needle trajectory definition. Based on the preplanning results and the final tumor’s position relative to the robotic system’s coordinates, each needle trajectory is defined.
- Needle insertion. The first needle in partially inserted up to a safe depth. A visual confirmation is required, using local CT scans, and thus, the needle trajectory is validated. In case the needle’s actual trajectory does not fit the predetermined one, the trajectory needs corrections. This means that the needle needs to be retracted and re-inserted using a new trajectory using the same target point into the tumor. If the needle trajectory is validated, another insertion depth is defined, and a second needle is taken from the needles rack and the entire procedure is repeated for each needle.

During the local CT scanning procedure, the robotic system must follow the CT couch (with the needle inserted into the patient’s body), with the same velocity, in the same direction, while the needle held by the MNID. The first needle is placed in the “middle” of the tumor, serving as a reference for the next needles, whose placement becomes more problematic, since, with each additional placed needle, the available workspace decreases.

After placing the required number of needles, the robotic system is removed, and the radiation seeds are placed using an afterloader [48,49]. Due to the medical prerequisites, this type of treatment requires the existence of an operating room, where the whole process must be carried out.

Risk estimation can be qualitative or quantitative [50]. Qualitative methods are usually used when quantitative data is unavailable or untrustworthy. In this case, a risk matrix is used, classifying risks into different zones (high, medium, low) and is the least used. The quantitative method indicates that the risk is the product of the probability of occurrence (PO) and the severity of the hazard (S).

The probability score PO is estimated as:

- Expected: 100;
- Quite possible: 80–99;
- Unusual, but possible: 50–79;
- Possible but unlikely: 30–49;
- Minor: 0–29.

The Severity, defined as the amount of damage or harm created by the hazard is classified as:

- Catastrophic: 100;
- Critical: 80–99;
- Serious: 60–79;
- Moderate: 30–59;
- Negligible: 0–29.

**Figure 7:**
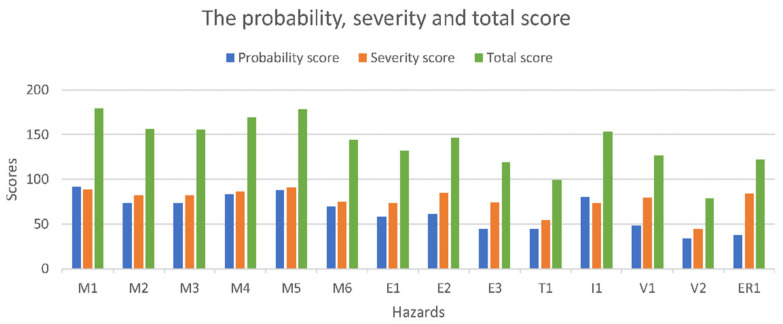
The risk assessment results.

The final score is obtained by adding the PO and S scores estimated by interviewing a team consisting of eight engineers with experience in the design of medical devices and two medical doctors, with a background in the minimally invasive treatment of HCC. Table 1 shows the PO score, while Table 2 presents the S score for the identified hazards, awarded for the needle insertion robotic system. Figure 7 presents the obtained results using a bar chart.

The highest probability score is M1 (needle deflection), while the most severe ones are M3 (collision with the CT) and M4 (the robotic system is unable to follow the CT couch). An evaluation scale used to evaluate the hazard scores has been developed as:

- Critical >180;
- High: 150–179;
- Moderate: 120–149;
- Minor: 50–119;
- Negligible: 0–49.

The final risk evaluation is performed in Table 3.

According to the presented scaling, the hazards that need to be addressed within a risk reduction process are the Moderate and High risks. Eleven hazards qualify for this process, namely: M1, M2, M3, M4, M5, M6, E1, E2, I1, V1, and ER3. During the MNID design and its integration within the KUKA iiwa robot, all hazards have been addressed, but the eleven hazards have become a special concern.

Table 4 presents the measures taken to reduce the risks pointed out in Table 3.

### 2.4. Failure Mode and Effect Analysis of the MNID

Failure Modes and Effects Analysis (FMEA) is a structured approach used to identify, analyze, and take the required measures to prevent issues related to the functionality of a product or process. The final goal is to yield a set of design, maintenance, or working conditions which would decrease the occurrence probability of unwanted events that would deter the device or process from the normal working way [51]. Ideally, FMEA should be performed during the design stage (as it is the case with the MNID), but even if applied on existing products or procedures, it will prove its benefits. The scope of FEMA is to:

- Identify the subsystems of the selected system (if the case);
- Analyze the main functions of the components;
- Identify the breakdown modes of each element performing the MNID functions, their potential effect, cause and the means to resolve the issues and avoid the negative results;
- Assess the identified hazards in terms of severity, occurrence and detection and calculate the Risk Priority Number (RPN = severity × occurrence × detection scores).

The potential failure mode indicates how the system may fail before it accomplishes the desired task. In our case, most of the function’s failure will somehow lead to an inaccurate placement of the needle within the patient’s body, which may have severe consequences. All these failures are potential, meaning that they may happen and not that they will happen, which means that there is a probability of occurrence (O). An immediate effect is generated, and its impact should be quantified (severity—S) based on an evaluation, with a more severe impact being awarded a higher score. Since most of the effects are strictly related to the patient’s health and safety, all these are treated as critical and must be addressed as such. Not all failures can be identified in due time (before the system’s breakdown), which is why this aspect must be quantified, with a larger amount being awarded in the case of reduced detectability (D).

Table 5 presents the process of the FMEA for the MNID, using a literal description for the described phases.

The classical FMEA approach, with the S, O, D scoring based on interviewing specialists with experience in the field, is a still a popular choice to assess the failure modes of products or processes, mainly due to the excellent provided results. Nevertheless, there are cases and situations when the practical results are ambiguous, yielding an unclear path for the design engineers. One can easily imagine that the same RPN score can be obtained for different combinations of S,O,D scores, while the actual breakdown risk is quite different. This is due to the fact that the importance of the scores (S,O,D) are very similar, at least theoretically, when, in real life, this is not the case. For this reason, we have decided to combine the FMEA approach with the fuzzy logic technique, which should yield a single output based on various input data [52].

Within this approach, the parameters S, O, D are fuzzified with specific membership functions, created using IF-THEN rules, which are further de-fuzzified to obtain the RPN value. Fuzzification implies the transformation of the input parameters into membership degree quantities, yielding a qualitative description of the parameter (in linguistic terms). This process has been achieved using the same 10 specialists, who provided the knowledge and the decision to establish the membership function degree for a certain variable. The fuzzy rules describe the risk level based on various combinations of the input parameters. De-fuzzification leads to the RPN single number using the output of the aggregated fuzzy set, so to obtain a crisp output.

**Figure 8:**
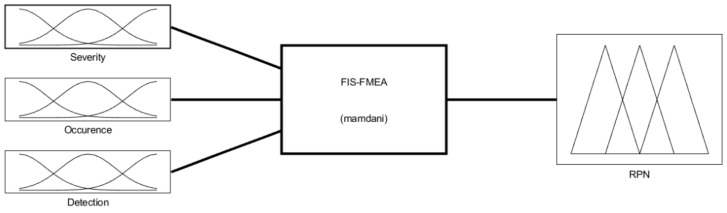
The Fuzzy Inference System for the FMEA (FIS-FMEA) provided by MATLAB.

**Figure 9:**
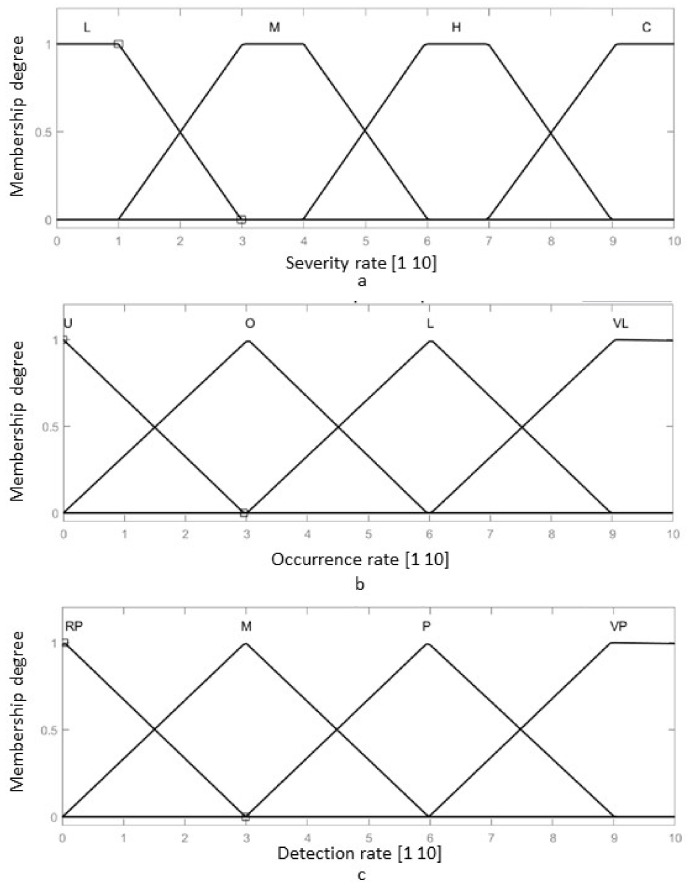
The membership function of the FIS-FMEA: (**a**) The input membership function for the Severity of the failure; (**b**) The input membership function for the Occurrence of the failure; (**c**) The input membership function for the Detection rate of the failure.

**Figure 10:**
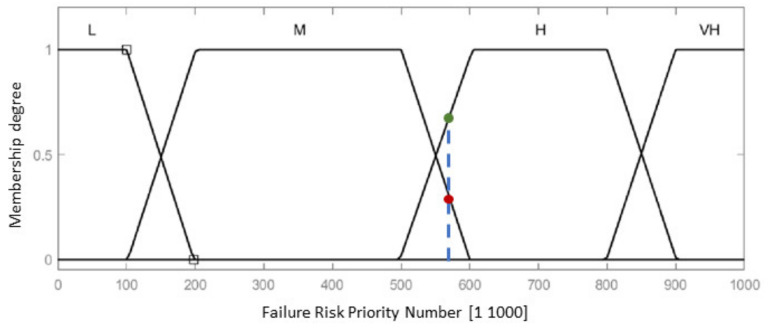
Output membership function for the Risk Priority Number of the failure.

The fuzzy-FMEA system has been implemented using the Mamdani FIS (Fuzzy Inference System) provided by MATLAB [53], Figure 8. The membership function for Severity, Occurrence and Detection are presented in Figure 9a–c, respectively. The input variables are scaled on a 1 to 10 scale using four levels as: Severity with Low (L), Moderate (M), High (H) and Critical); Occurrence with Unlikely (U), Occasional (O), Likely (L) and Very Likely (VL) and Detection with Remotely Probable (RP), Moderate (M), Probable (P) and Very Probable (VP). The output membership function of RPN is presented in Figure 10, while the numerical results for the RPN are presented in Table 6.

The membership functions for Severity have been modelled using a trapezoidal function, using Equation (1):

(1) $$trapezoid\left(x,a,b,c,d\right)=\left\{\begin{array}{cc} 0, & \;x\leq a\\ \frac{x-a}{b-a}, & a\leq x\leq b\\ 1, & b\leq x\leq c\\ \frac{d-x}{d-c}, & c\leq x\leq d\\ 0, & d\leq x \end{array}\right.,$$

where a represents the lower limit of the considered interval of values, b and c respectively the inferior and superior core values, while d is the superior limit of the interval of the membership for which the memberships functions degree is different from 0.

The membership functions of the Occurrence and Detection rates have been modeled using triangular functions, using Equation (2):

(2) $$triangle\left(x,m,n,p\right)=\left\{\begin{array}{cc} 0, & x\leq m\\ \frac{x-m}{n-m}, & m\leq x\leq n\\ \frac{p-x}{p-n}, & n\leq x\leq p\\ 0, & p\leq x \end{array}\right.,$$

where m represents the lower limit value of the interval, n is the core of the membership (maximum value of the of the interval) and p is the superior limit of the interval for which the membership functions degree is different than 0.

The RPN has been obtained using a set of 30 rules as follows:

I. IF Severity is H AND Occurrence is O AND Detection is M THEN RPN is H
II. IF Severity is H AND Occurrence is O AND Detection is VP THEN RPN is M
III. IF Severity is H AND Occurrence is L AND Detection is VP THEN RPN is M
IV. IF Severity is C AND Occurrence is L AND Detection is P THEN RPN is H
V. IF Severity is H AND Occurrence is VL AND Detection is P THEN RPN is H
VI. IF Severity is H AND Occurrence is O AND Detection is VP THEN RPN is L
VII. IF Severity is H AND Occurrence is O AND Detection is RP THEN RPN is VH
VIII. IF Severity is H AND Occurrence is VL AND Detection is VP THEN RPN is M
IX. IF Severity is M AND Occurrence is L AND Detection is RP THEN RPN is VH
X. IF Severity is M AND Occurrence is L AND Detection is VP THEN RPN is L
XI. IF Severity is L AND Occurrence is VL AND Detection is VP THEN RPN is L
XII. IF Severity is L AND Occurrence is L AND Detection is RP THEN RPN is VH
XIII. IF Severity is L AND Occurrence is L AND Detection is M THEN RPN is H
XIV. IF Severity is L AND Occurrence is L AND Detection is P THEN RPN is M
XV. IF Severity is L AND Occurrence is VL AND Detection is VP THEN RPN is L
XVI. IF Severity is M AND Occurrence is L AND Detection is RP THEN RPN is VH
XVII. IF Severity is M AND Occurrence is L AND Detection is M THEN RPN is VH
XVIII. IF Severity is M AND Occurrence is L AND Detection is P THEN RPN is H
XIX. IF Severity is M AND Occurrence is L AND Detection is VP THEN RPN is L
XX. IF Severity is C AND Occurrence is L AND Detection is VP THEN RPN is M
XXI. IF Severity is C AND Occurrence is L AND Detection is P THEN RPN is VH
XXII. IF Severity is C AND Occurrence is L AND Detection is VP THEN RPN is M
XXIII. IF Severity is C AND Occurrence is VL AND Detection is P THEN RPN is H
XXIV. IF Severity is C AND Occurrence is L AND Detection is RP THEN RPN is VH
XXV. IF Severity is L AND Occurrence is O AND Detection is VP THEN RPN is L
XXVI. IF Severity is H AND Occurrence is O AND Detection is P THEN RPN is H
XXVII. IF Severity is M AND Occurrence is VL AND Detection is RP THEN RPN is VH
XXVIII. IF Severity is H AND Occurrence is U AND Detection is VP THEN RPN is M
XXIX. IF Severity is H AND Occurrence is U AND Detection is VP THEN RPN is M
XXX. IF Severity is H AND Occurrence is O AND Detection is RP THEN RPN is H

The formulation of the fuzzy rules (“If—Then” rule) has been achieved considering that most of the failures are practically “severe”, so that the Detection of the failure becomes the decisive factor within the value of RPN. Thus, if the Detection is RP and the Severity or Occurrence are M, respectively L, the RPN score is VH.

**Figure 11:**
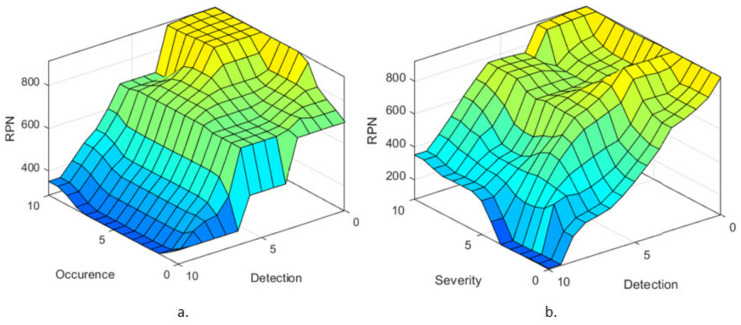
Failure level surface with respect to (**a**) Occurrence and Detection membership functions and (**b**) Severity and Detection membership functions.

Using the previously discussed membership functions and the IF-THEN rules, the surface distribution of the RPN considering the Occurrence and Detection rates is presented in Figure 11a, while the surface distribution of the RPN considering the Severity and Detection rates is presented in Figure 11b. Ideally, the rules surfaces should have no vertical lines, being as smooth as possible, showing that the transition from one failure level to the next is achieved fluidly. Furthermore, such a surface rule shows that for each input there is a determined output, whether it is a value embedded in the database or a null response. Both Figure 11a,b. lack such vertical lines, which validates the developed FIS.

**Figure 12:**
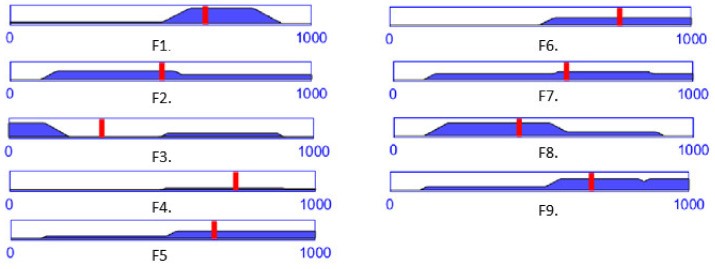
The RPN output level for each failure mode computed with the FIS-FMEA using the data in Table 7.

The same team of specialists has been asked to provide an estimation on a 1 to 10 scale for the failure modes in Table 5, the mean value being presented in Table 7. Figure 12 presents RPN output membership functions values for the input values into the developed FIS-FMEA shown in Table 7, for each identified failure mode.

According to Table 6 and Table 7, a RPN score of 648 is entirely High (for the failure mode F1), while a RPN score of 578 (for the failure mode F7) is 78% High and 22% Medium (Figure 10, the blue dotted line intersecting the trapeze which indicates the membership function degree), which directly influences the measures (regarding the mechanical design, medical protocol, control system and costs) taken to avoid these failures modes.

## 3. Results

The set of prioritized medical requirements, of measures directed to reduce the risks involved by such an exigent operation and to prevent the identified possible failure modes have further resulted in the development of a robotic instrument for the minimally invasive treatment of HCC. The MNID development is described in this section in terms of mechanical design and control system, to fit the previous section output. Validation tests in medical environment using a human phantom and a ballistic gel liver are also presented, targeting mainly the needle placement accuracy within the specified environment, which is representative for the overall efficiency of the proposed solution.

### 3.1. The Robotic System Design

**Figure 13:**
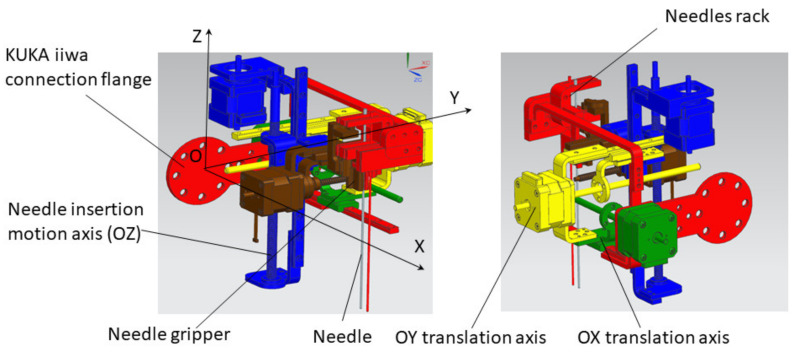
The Multiple Needle Insertion Device CAD model [56].

**Figure 14:**
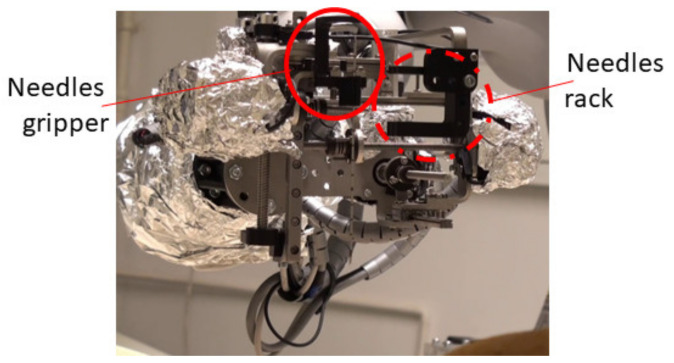
The experimental model of MNID.

Following the concept in Figure 4, a needle insertion device capable of handling multiple needles and positioned at the insertion site using the KUKA iiwa LBR R800 collaborative robot has been designed and is presented in Figure 13. The MNID has the following main components: the needle gripper, the needle insertion axis, and the OX and OY motion axis of the Gantry architecture, each one consisting of an actuator, a screw–nut mechanism, and a rail-and-sledge linear guide [54]. The experimental model of the MNID is presented in Figure 14, mounted on the KUKA iiwa flange, with the motors covered with aluminum foil to protect them during the CT scanning and ready for the performance of validation tests. The actuators are stepper motors with trapezoidal threaded shaft for increased compactness, from Nanotec [55].

**Figure 15:**
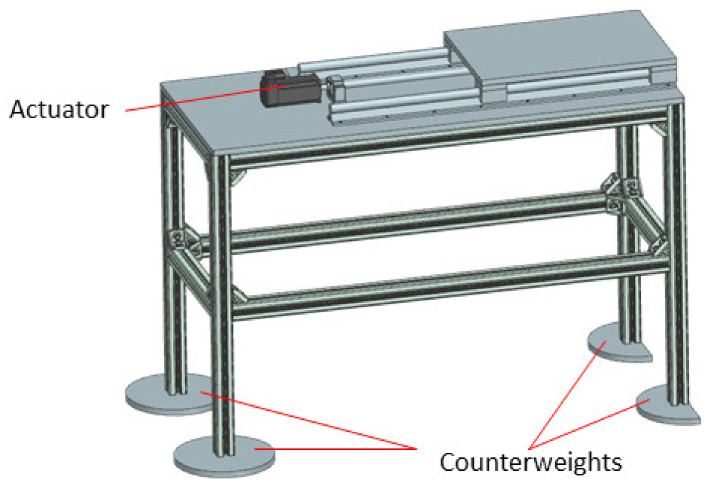
The external motion axis to follow the CT couch.

As already mentioned in Section 2.3, the robotic system needs to follow the CT couch during the scanning procedure, which is essential to determine if the needle is on the correct trajectory within the patient’s body. For this purpose, a distance sensor has been considered, namely the IFM 01D100 [57], which is used to accurately track the CT couch. Although the KUKA iiwa should be able to follow, at least theoretically, the CT couch alone, the required motion trajectory should be carefully studied and before starting the procedure and make sure it is completely included within the robot’s workspace. This is a difficult procedure and would require significant resources. To avoid such complications, a 1 DoF external motion axis to drive the robotic system (KUKA iiwa and the MNID attached) has been designed and is presented in Figure 15. It is an aluminum alloy profiles construction, counterweights being designed and added at each of the table’s legs to ensure the system’s stability.

### 3.2. The Control System

**Figure 16:**
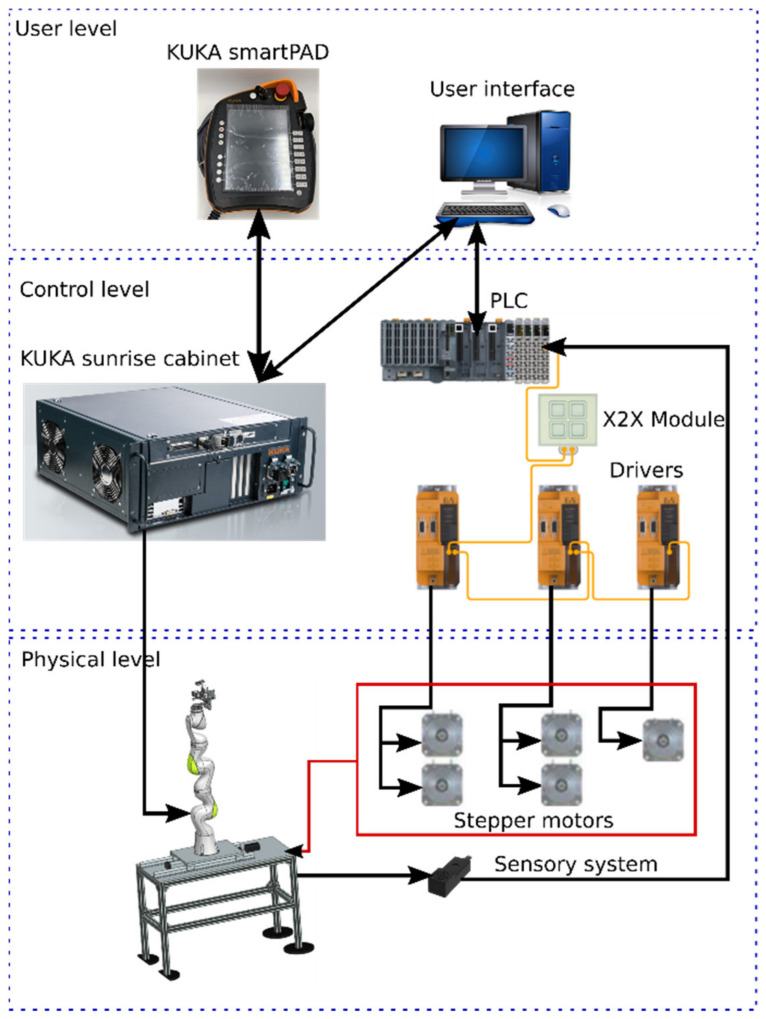
The control architecture of the robotic system consisting of the KUKA iiwa, MNID, and 1 DoF external axis.

It is a hybrid system, where the KUKA iiwa is driven by the sunrise cabinet, the MNID actuators and the 1 DoF axis being separately controlled using a PLC from B&R Automation, namely the X20CP1586 [58]. The control scheme is presented in Figure 16.

The control architecture is structured on three levels:

- User level, consisting of the KUKA smartPAD and the User Interface (PC);
- Control level, consisting of the KUKA Sunrise Cabinet and the PLC. The drivers used for the Nanotec stepper motors are also from B&R Automation, namely 80SD100XD.C011-01, each one being able to drive 2 motors;
- Physical level, consisting of the KUKA iiwa robot, the MNID and the 1 DoF axis, their actuators, and the sensory system. The latter consists of the proximity sensors used for the initialization procedure of each motion axis (and as stroke limiters) and the distance sensor from IFM.

**Figure 17:**
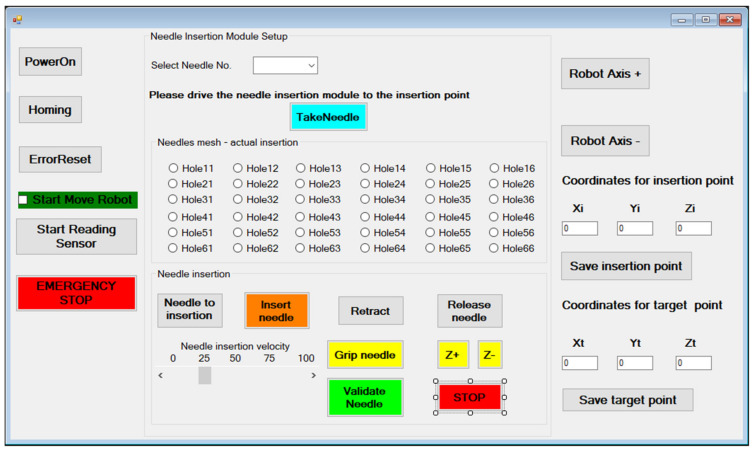
The MNID User Interface.

The User Interface (Figure 17) is used to control the MNID and the external axis once the KUKA iiwa has positioned the MNID at the insertion site, within the CT bore just above the patient’s abdominal cavity (close to the liver).

The “PowerOn” and “Homing” buttons perform the main steps of the initialization procedure. Needles are numbered within the rack and their extraction order is predetermined, but the operator is still given the chance to change the order. However, this is implying a very high risk. After gripping the needle (“Gripe needle” button), it is removed from the needle rack (the “TakeNeedle” button) and the operator can choose the insertion position. The MNID needle insertion workspace is segmented using 36 positions encoded $N_{ij}$ based on their position on the planar matrix. Initially, a physical (3D printed) sieve with these holes has been designed, but during the first tests it has been removed due to the tight space within the CT bore. After selecting the desired position (hole), the needle is driven at the insertion point (“Needle to insertion” button). Once the needle position is acknowledged, the needle insertion procedure begins (the “Insert needle” button). The needle insertion velocity must be selected before starting the actual insertion procedure. According to the protocol, the needle position is constantly verified through using local CT scans at selected time intervals. Before the scanning procedure, the “Start Reading Sensor” button must be pushed to begin reading the distance sensor signal. Once the CT couch starts moving, the 1 DoF axis will follow it. Before the scanning, the needle insertion is stopped (the “STOP” button), and the needle is held crisply in position. Within the performed tests, the needle has been inserted within the CT bore the scanning being performed while the CT couch has retracted out from the CT. A decision process follows with the following outputs: continue the insertion or retract the needle (the “Retract” button) to insert it using a different hole. If the needle reaches successfully (or within acceptable limits) the target point, the needle position is validated (“Validate needle” button), and the needle can be released (the “Release needle” button). The operator can also move the needle on the current selected trajectory using the “Z+” or “Z-” buttons. The needle validation also means that the current hole is now occupied, turning it red and thus becoming unselective, so it prevents the user to use it again for further needles. The “Release needle” button drives the gripper back to the initial position, to the one before the needle has been inserted. The procedure can now continue with the selection of the next needle.

Alternatively, the user can use the coordinates of the Insertion point (at the level of the abdominal cavity of the patient) and Target point (within the lesion in the liver), the Xi, Yi, Zi and Xt, Yt and Zt to place the needle. The MNID position within the CT bore is usually achieved through a motion combination of the KUKA iiwa and the 1 DoF external axis (using the “User Axis+” and “User Axis-” buttons), which is used, as already mentioned, to increase the total workspace of the robotic system.

### 3.3. Validation Tests

**Figure 18:**
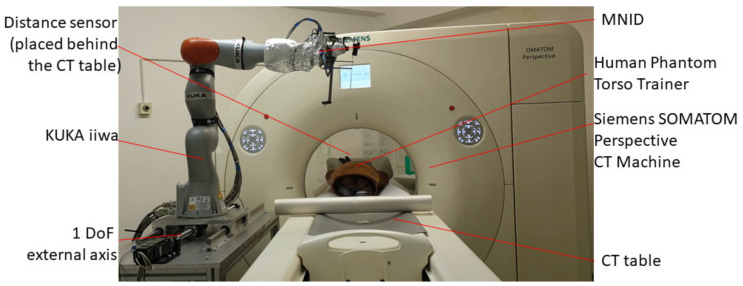
The robotic system in medical environment.

**Figure 19:**
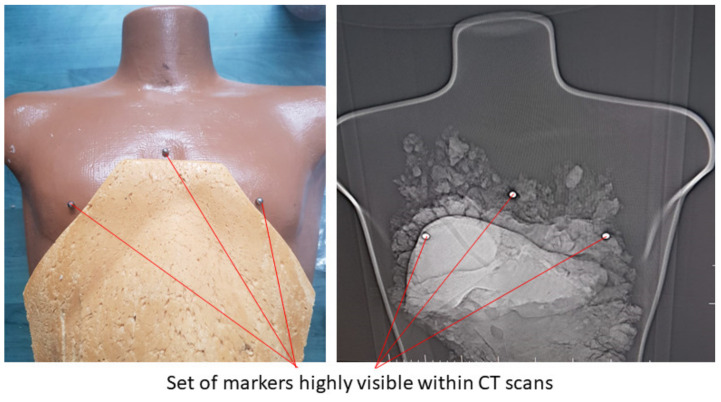
The set of markers used within the registration procedure of the tumors in the robotic system’s coordinates.

Experimental tests to validate the MNID and the robotic system have been performed at the “Regional Institute of Gastroenterology and Hepatology Prof. Dr. Octavian Fodor” from Cluj-Napoca, Romania. The experimental model in the medical environment is presented in Figure 17. The layout mostly replicates the elements presented in Figure 4, namely: the KUKA iiwa, the MNID, the 1 DoF external axis, the CT machine with the CT couch, a human phantom and the IFM distance sensor (which is not visible in Figure 18 since it has been placed behind the CT couch). The registration procedure, according to the protocol presented in Figure 5, is performed using a set of markers highly visible within the CT scans, Figure 19.

**Figure 20:**
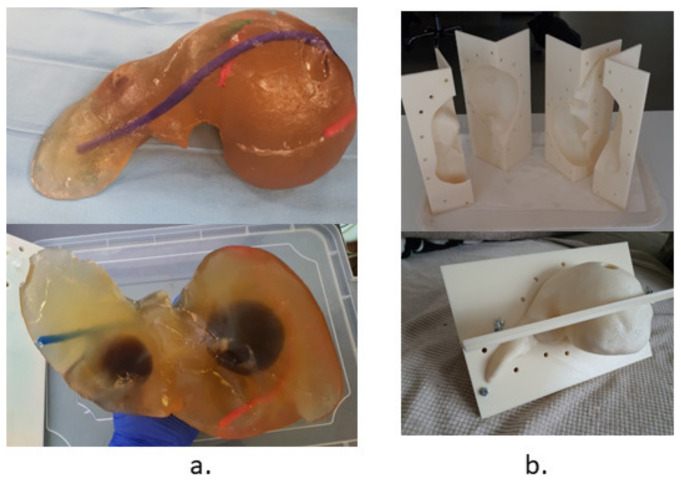
The testing and validation liver: (**a**) The ballistic gel liver with blood vessels and tumors. (**b**) The 3D printed mold.

**Figure 21:**
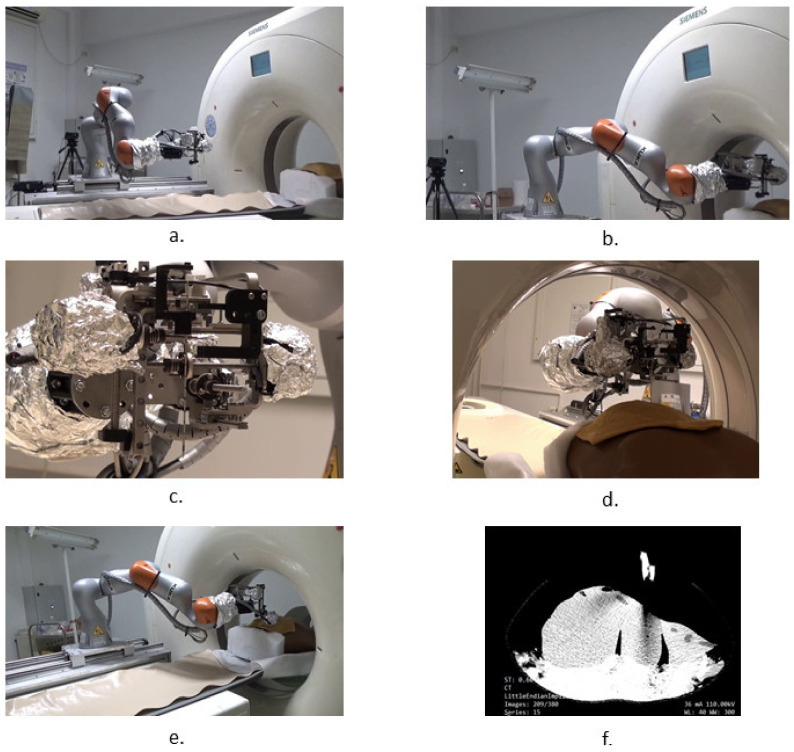
Snapshots during the robotic system validation tests: (**a**) MNID driven to the insertion site, above the patient; (**b**) Registration process; (**c**) Needle gripping; (**d**) Needle insertion; (**e**) CT scanning procedure; (**f**) CT image with the liver and needles.

The validation tests have been performed using a human-size ballistic gel liver, having the main blood vessels (hepatic artery and vein) manually positioned as well as a set of tumors simulated and precisely positioned within it, as illustrated in Figure 20a. The liver has been obtained following the 3D scan of a human liver and the 3D printing of a mold, as in Figure 20b. Figure 21 shows a series of snapshots for the robotic system position inside the CT, needle gripping, needle insertion, and scanning during validation tests.

**Figure 22:**
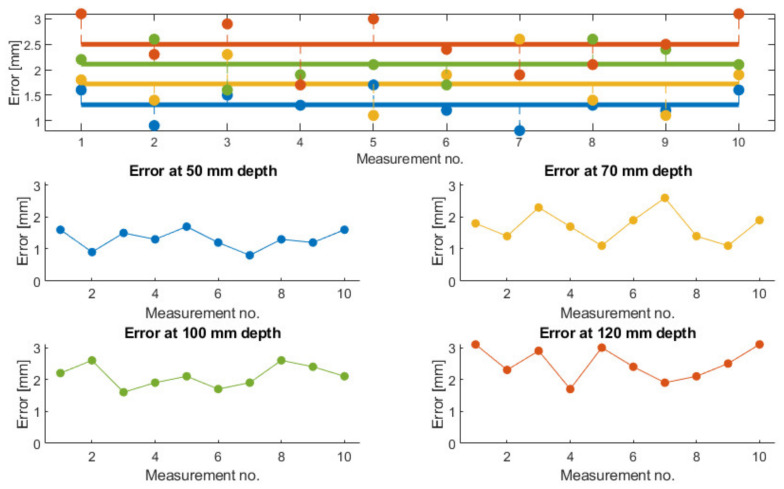
Measured needle placement error during validation tests.

A set of 10 measurements of the needle placement procedure have been taken for four different target points, located at determined depths (50, 70, 100 and 120 mm). The measurements have considered only the final needle position and compared it with the initial calculated position. The results are presented in Figure 22. The mean error for the measured depths is: 1.3, 1.72, 2.11, and 2.5 mm, while the overall mean error is 1.91 mm. The RMSE (root mean square error) for each depth is: 1.34, 1.78, 2.13, and 2.54 mm, while the mean standard error is 0.411 mm.

## 4. Discussion

Current standards regarding the development of surgical robotic systems do not clearly specify a strategy in the design of a safe and efficient system for the minimally invasive treatment of deeply located tumors, such as the HCC. The paper proposes a method of designing a modular robotic system that provides a solution not only to the medical requirements, but also targets the overall workflow within a cancer treatment hospital.

Thus, starting from the general requirements, some of which being presented in ISO 11608 or IEC 60601, a robotic needle insertion device has been proposed. The combination of a commercially available robotic arm, namely the KUKA iiwa LBR R800, which is quasi-identical with its sibling, the KUKA LBR Med, which implements all standards required by an operation room, and a custom designed needle insertion device has multiple benefits. The KUKA iiwa is a lightweight robot, with proven collaborative features that can be used in any medical facility, if correctly and efficiently programmed. The MNID has been designed to fit the payload capabilities of the KUKA iiwa (it weighs 3.09 kg), which means that all collaborative features of the robot remain active, thus any collision with elements from the medical environment are easily detected and the negative effects avoided. The MNID has been designed considering the high spatial constraints imposed by the currently in use CT bores, since the overall dimensions of the MNID are: 220 × 210 × 200 mm (L × l × h). This means that the robotic system (KUKA iiwa + MNID) fits most diameters of classical CT bores, considering a medium sized patient. The same KUKA iiwa provides enough accuracy for the position and orientation of the robot flange within the CT bore, so coupled with the extra-workspace provided by the 1 DoF external axis it provides an accurate position of the MNID at the insertion site. The Gantry architecture of the MNID implemented using pretensioned trapezoidal nuts ensures the positional accuracy of the needles at the insertion site and during the insertion procedure. The solid stainless-steel structure of the MNID ensures the required stiffness, as observed during validation tests.

Due to its rather complicated mechanical structure, the only way to ensure the sterilization of the MNID is to use sterilizable motors. Within the current development stage of the MNID, which aimed the validation on phantom molds did not impose the use of sterilizable motors the experimental model being built with their standard counterparts. Patient breathing will be investigated and embedded in the control loop within the next development stages, thus a controlled patient breathing which allows the implementation of the motion gating approach will also be sought soon. Last, but not least, the modularity of the robotic system offers the possibility to develop and use robotic platforms within the oncology clinics, by utilizing interchangeable medical instruments and devices, suitable for various applications and treatment methods, ranging from minimally invasive surgery to accurate needle placement for the treatment of different maladies affecting different organs of the patients.

Benefiting from the input of a team of experienced engineers and physicians, a set of hazards have been identified and analyzed to reduce the accident risks of an already very risky procedure. Needle deflection is one of the most dangerous hazards, because it is highly unpredictable (at least with straight needles) and difficult to avoid. Most of the times, the needle must be removed and reinserted again on a different trajectory. A practical solution might be to perform small incisions within the tissue before the needle penetration, but within the tight space of the CT bore, this is almost impossible. Most of the other hazards have been avoided with proper design. M2, collision with the patient’s body and M3, collision with the CT, are very probable, again due to the very tight space. Of course, M3 is more dangerous, since it might alter the rather delicate structure of the MNID, even if the KUKA iiwa torque sensors immediately detect the collision. This is why a certain safety distance has been considered during the validation tests. During these tests, the gripper has performed a faulty gripping only once (out of more than 100 gripping tests), caused by the needle’s position change within the needle rack, which is why we have considered it an accident with a very rare occurrence. Some of the hazards (e.g., E1, E3, I1, ER1) did not happen at all during the performed tests.

The paper also presents a fuzzy logic-based Failure Mode and Effect Analysis targeting an improved design of the MNID. After identifying the failure modes of the robotic system, FIS has been used to estimate the global risk of the identified failures, based on the input variables: Severity, Occurrence and Detection. A trapezoidal membership function has been defined for the Severity, while triangle membership functions have been defined for Occurrence and Detection. The inference rules of the FIS-FMEA have been established and graphically represented, based on the experience of the team of experts active within the current research. Based on their input, the mean scores of each input variable for the determined failure modes have been calculated and used within the defined FIS-FMEA to obtain the Risk Priority Number for the nine failure modes. The FIS-FMEA output has been used to upgrade the design of the MNID and the whole robotic system, although at least some of the issues have been addressed in a certain measure previously. Thus, the needles have been numbered and their extraction from the rack performed in a predetermined order. This has not been an issue during the validation tests. The gripper has been designed to grip the needles tested within our application: Franseen tip and 2.1 mm in diameter. The gripper jaws have been modified and now their length is 20 mm for a firm grip. The gripper’s jaws have been modified several times during laboratory tests, especially due to the failure’s modes characterizing the F7-F9 functions, leading to their current design, by implementing the recommended action in Table 5. One jaw has an upper blocking element used to prevent the needle from slipping along the OZ axis during the insertion. The same element can be further used do install a force sensor, which could provide some information by recording the insertion forces. During the tests using ballistic gel [59], these forces have proven to be at a very low level, so a decision based on this data would not be very accurate at this stage of development. The stroke lengths have proven to be just enough to safely perform the tests.

The accuracy measurements show an overall mean error of 1.91 mm. This value is better than some of the results published by other researchers (also CT guided needle insertion, e.g., [17]), but our results are obtained only on ballistic gel and not animal tissue, which partially explains the situation.

Although needle insertion is a highly studied process in medical robotics, most of the devices have been designed to be used and visually guided by an ultrasound probe, either percutaneously or intraoperatory. Most worldwide research results are centered on the development of robotic systems for brachytherapy procedures aided by visual guidance from the MRI machines, but mostly for the prostate cancer treatment, since the risks are vastly reduced. A very small percentage of the research in the field has been targeted towards the CT-guided procedures (as observed within the state of the art study), maybe because of three reasons: the lack of real-time control (thus very high risks of puncturing unwanted tissue, e.g., important blood vessels), the irradiation of the patient (and possibly the operator), and because this procedure requires an operating room, a high cost investment, which is difficult to be recovered in a short time for smaller hospitals.

## 5. Conclusions

The paper proposes a design methodology for the development of a robotic needle insertion device to be used in conjunction with a commercially available robotic arm as a positioning system in a modular open architecture. The development has been performed based on medical specific requirements, a risk assessment of the main identified hazards and a fuzzy logic based FMEA system.

The team of engineering experts and physicians who assisted the validation tests in medical environment accepted the developed robotic system and provided positive feedback that is an incentive in considering the next steps in the development of the MNID.

Although the robotic system has passed the initial functionality test, future work is mandatory to improve the performances of the system. For example, the registration needs to be improved to ensure a higher placement accuracy. Further attention needs to be directed towards the User Interface, making it simpler and more intuitive. The 3D printed parts of the experimental model will have to be replaced with sterilizable metal components, as it will also be done with the stepper motors. Considering the high-risk application and the large number of constraints, the initial results are a proof that the system can become a viable solution for the treatment of inoperable liver cancer.

## Figures and Tables

**Table 1:** The Probability of Occurrence score.

Hazard	En1	En2	En3	En4	En5	En6	En7	En8	MD1	MD2	Mean Value
M1	90	95	90	95	85	90	95	90	90	90	91
M2	70	80	90	80	75	65	70	75	65	70	74
M3	65	80	90	80	75	65	70	75	65	70	73.5
M4	85	85	90	85	90	95	75	80	70	75	83
M5	90	85	95	80	95	90	80	85	88	92	88
M6	70	75	60	65	60	70	80	75	70	70	69.5
E1	50	60	60	70	75	50	55	65	50	50	58.5
E2	50	60	65	55	58	65	70	75	55	60	61.3
E3	40	45	40	50	40	45	60	55	40	40	45.5
T1	45	35	40	45	55	45	50	55	35	40	44.5
I1	100	100	95	100	95	90	95	100	100	100	97.5
V1	50	55	60	45	50	65	40	35	40	40	48
V2	35	30	40	30	35	30	40	35	30	30	33.5
ER1	30	40	45	40	45	30	35	40	35	35	37.5

**Table 2:** The Severity score.

Hazard	En1	En2	En3	En4	En5	En6	En7	En8	MD1	MD2	Mean Value
M1	90	95	85	90	80	85	80	90	95	95	88.5
M2	85	85	90	85	80	90	80	75	75	75	82
M3	85	85	90	85	80	90	80	75	75	75	82
M4	100	100	100	100	100	95	100	100	100	100	99.5
M5	100	95	100	95	100	90	100	95	95	95	96.5
M6	80	85	75	70	85	70	65	75	75	70	75
E1	80	85	80	70	75	70	65	70	75	70	74
E2	100	95	100	90	95	100	95	90	95	100	96
E3	75	70	75	70	85	80	80	75	70	65	74.5
T1	60	55	50	55	55	50	60	55	55	50	54.5
I1	70	75	70	80	80	85	70	70	65	70	73.5
V1	80	85	80	90	85	70	80	85	70	65	79
V2	50	45	45	40	50	55	40	45	40	40	45
ER1	90	95	80	95	85	75	85	80	80	80	84.5

**Table 3:** Risk evaluation of the identified hazards.

Hazard	Score	Evaluation
M1	179.5	High
M2	156	High
M3	155.5	High
M4	169.5	High
M5	178.5	High
M6	144.5	Moderate
E1	132.5	Moderate
E2	146.3	Moderate
E3	119.5	Minor
T1	99	Minor
I1	153.5	Moderate
V1	127	Moderate
V2	78.5	Minor
ER1	122	Moderate

**Table 4:** Measures to reduce the hazards risks.

Hazard	Measure Taken to Reduce the Risk
M1	Needle deflection is almost impossible to avoid but must be kept under certain limits. This is the main reason why the expected accuracy (~2.5 mm) is rather poor within these applications. This hazard is strongly related to the procedure control flow and the only way to avoid the negative effects of deflection is to carefully monitor the needle trajectory between two consecutive scans and decide if it still fits the required outcome in terms of final position within the tumor (if the radiation time or intensity can be adjusted accordingly) or if it hits vital tissue (e.g., important blood vessels), case in which it has to be removed and the trajectory adjusted.
M2	Proximity sensors have been mounted on the MNID and the stroke of each axis is strictly monitored. The torques within the KUKA iiwa are also be monitored. Joint velocities are limited when the MNID approaches the patient.
M3	Since KUKA iiwa is a collaborative robot, the torques are strictly monitored. Limit the ranges of motion of each axis and use proximity sensors.
M4	An additional motion axis has been installed and programmed to use the signal of a distance sensor measuring the real-time displacement of the CT couch within the CT bore.
M5	The needle rack has been designed to firmly hold up to 6 needles using elastic elements. The needle locations are numbered and sufficiently spaced. An artificial ventilation system will be used to strictly monitor the patient’s breathing, which allows the implementation of the motion gating strategy.
M6	The gripper has been custom designed to grip the needles using a large area. Stroke limiters have been installed.
E1	Low voltage components have been used and the proper regulated protection of the system has been installed.
E2	A strict protocol has been developed, in which all functions of the robotic system are tested within the initialization phase. Signal monitoring is strictly monitored. Proper regulated protection has been used.
E3	Use proper regulated protection for the system.
T1	Avoid using parts that would create heat in contact with the patient. Avoid unnecessary contact with the patient in general.
I1	CT scanning implies irradiation with X-rays. The focus here is to avoid unnecessary irradiation (e.g., fewer CT scans) and the strict delimitation of the CT scan range. Nevertheless, irradiation within this kind of procedure cannot be avoided.
V1	Avoid resonance. Check for loose parts.
V2	Check for loose parts. Use low friction materials (e.g., stainless steel screw with brass nut).
ER1	Firmly hold the patient in the right position on the CT couch. Constantly check the tumor position.

**Table 5:** Failure Modes and Effects Analysis (FMEA) for MNID.

Code	Function	Potential Failure Mode	Potential Failure Effect	Potential Cause	Recommended Actions
F1	Needle gripping	Wrong needle is gripped	The insertion order may be disrupted. Other needles may fall from the rack	Wrong numbering, rack position changed, needle missing from the rack	Before starting the procedure check that all needles are in place, in the correct order.
F2	Needle gripping	Inaccurate positioning	The needle does not reach the target point	The needle may move inside the gripper during insertion	Design the gripper to firmly grip the needles using specific dimension grooves.
F3	Needle positioning	Reach the end of motion range	The needle does not reach the target point	Not enough stroke Lack of stroke limiters	Design properly the stroke lengths. Install stroke limiters
F4	Needle positioning	Inaccurate positioning	The needle does not reach the target point	Play within the screw-nut mechanism	Use preloaded nuts. Check them after each 5 procedures
F5	Needle insertion	Inaccurate positioning	The needle does not reach the target point	Needle slips inside the gripper	Design the gripper to block the slipping tendency
F6	Needle insertion	Patient’s liver hemorrhage	Unexpected blood loss	Needle deflects from the imposed trajectory	Install force sensor to detect out of range insertion forces.
F7	Needle insertion	Inaccurate positioning	Unexpected blood loss. The needle does not reach the target point	Current needle collides with previous inserted needles	Design the gripper to avoid accidental collisions. Use parallel trajectories. Insert first needle in “the middle of the tumor”
F8	Needle insertion	Inaccurate positioning	The needle reaches the tumor, but not the imposed target point	Needle deflects from the imposed trajectory	Choose one of the following: remove and reinsert needle; recalculate the other needles trajectories; recalculate dosimetry
F9	Needle retraction	Inaccurate positioning	After insertion, the needles move from the targeted lesion	The gripper collides with the previous inserted needles	Design the gripper jaws in a slight conical form. Install stroke limiters to control the gripper opening

**Table 6:** The Output membership function numerical results for the RPN.

RPN Value	Failure Risk Linguistic Variable
0–200	Low
100–600	Moderate
500–900	High
800–1000	Very High

**Table 7:** The S, O, and D scores awarded by the team of specialists.

Failure Mode	Severity	Occurrence	Detection	RPN Value
F1	6.3	3.1	6.2	648
F2	8.1	6.4	8.1	503
F3	3.2	5.5	8.4	305
F4	7.1	5.7	4.7	739
F5	7.9	7.2	6.4	669
F6	9.8	5.8	4.3	763
F7	9.1	7.9	7.5	578
F8	6.2	9.1	8.3	419
F9	8.4	6.9	6.6	674

## Data Availability

The data presented in this study are available on request from the corresponding author.
